# A New *Enterobacter cloacae* Bacteriophage EC151 Encodes the Deazaguanine DNA Modification Pathway and Represents a New Genus within the Siphoviridae Family

**DOI:** 10.3390/v13071372

**Published:** 2021-07-15

**Authors:** Vera Morozova, Ghadeer Jdeed, Yuliya Kozlova, Igor Babkin, Artem Tikunov, Nina Tikunova

**Affiliations:** 1 Laboratory of Molecular Microbiology, Institute of Chemical Biology and Fundamental Medicine, Siberian Branch of Russian Academy of Sciences, 630090 Novosibirsk, Russia; morozova@niboch.nsc.ru (V.M.); ghadeerjdeed@outlook.com (G.J.); ulona@ngs.ru (Y.K.); i_babkin@mail.ru (I.B.); arttik@mail.ru (A.T.); 2 Department of Natural Sciences, Novosibirsk State University, 630090 Novosibirsk, Russia

**Keywords:** *Enterobacter* phage, *Seuratvirus*, *Nonagvirus*, deazaguanine modification pathway, preQ_0_ transporter

## Abstract

A novel *Enterobacter cloacae* phage, EC151, was isolated and characterized. Electron microscopy revealed that EC151 has a siphovirus-like virion morphology. The EC151 nucleotide sequence shows limited similarity to other phage genomes deposited in the NCBI GenBank database. The size of the EC151 genome is 60,753 bp and contains 58 putative genes. Thirty-nine of them encode proteins of predicted function, 18 are defined as hypothetical proteins, and one ORF identifies as the tRNA-Ser-GCT-encoding gene. Six ORFs were predicted to be members of the deazaguanine DNA modification pathway, including the preQ_0_ transporter. Comparative proteomic phylogenetic analysis revealed that phage EC151 represents a distinct branch within a group of sequences containing clades formed by members of the *Seuratvirus, Nonagvirus*, and *Vidquintavirus* genera. In addition, the EC151 genome showed gene synteny typical of the *Seuratvirus, Nonagvirus,* and *Nipunavirus* phages. The average genetic distances of EC151/*Seuratvirus*, EC151/*Nonagvirus*, and EC151/*Vidquintavirus* are approximately equal to those between the *Seuratvirus, Nonagvirus,* and *Vidquintavirus* genera (~0.7 substitutions per site). Therefore, EC151 may represent a novel genus within the *Siphoviridae* family. The origin of the deazaguanine DNA modification pathway in the EC151 genome can be traced to *Escherichia* phages from the *Seuratvirus* genus.

## 1. Introduction

The *Enterobacteriaceae* family includes anaerobic, motile, Gram-negative rods belonging to the Enterobacterales order, Gammaproteobacteria. This family comprises many genera (*Citrobacter*, *Enterobacter*, *Escherichia*, *Klebsiella*, *Proteus*, *Salmonella*, *Shigella*, *Yersinia,* etc.) containing both environmental, medical, and agriculturally important bacteria. Currently, the genus *Enterobacter* contains 22 species [1,2] and is associated with a variety of environmental habitats. These bacteria are found in water, soil and are phytopathogens for various plant species [2]. *Enterobacter* spp. are also found as a part of the normal animal and human gut microbiota. Among these bacteria, only certain subspecies/species have been associated with hospital-acquired infections and outbreaks. *E. cloacae* and *E. hormaechei* represent the most frequently isolated species described in clinical infections [3]. Since many medically important strains of enterobacteria are antibiotic resistant, lytic phages against such bacteria are of great interest.

There are thirty-six prototype *Enterobacter* phage genomes deposited in the NCBI GenBank database (www.ncbi.nlm.nih.gov/genomes/GenomesGroup accessed on 1 June 2021). Seventeen *Enterobacter* phages possess the myovirus morphotype: sixteen of them belong to the *Myoviridae*, and one, to the *Ackermannviridae* family. Eight and three phages are members of the *Autographiviridae* and *Podoviridae* families, respectively. The remaining eight phages possessed the siphovirus morphotype and were defined as *Siphoviridae* (six phages) and *Drexlerviridae* (two phages) family members.

It has been previously found that some enterobacteria phages contain genes responsible for the biosynthesis of queuosine [4,5,6,7]. Queuosine biosynthesis pathway produces modified nucleoside derivatives of guanosine (queuosine (Q) in bacteria and archaeosine (G+) in archaea). 7-Cyano-7-deazaguanine (PreQ_0_), an intermediate in both the Q and G+ pathways, is synthesized from GTP by four enzymes: FolE, QueC, QueD, and QueE. Both Q and G+ were thought to exist exclusively in tRNA molecules. Queuosine is known to be involved in the modification of cognate tRNAs (histidine, asparagine, aspartic acid, and tyrosine) by replacing guanine in the wobble position (position 34), and it was shown to improve decoding accuracy in protein synthesis [8].

Recently, it has been found that queuosine biosynthesis genes in phage genomes are responsible for 7-deazaguanine derivative insertion into phage DNA [8,9,10]. 7-Deazaguanine modifications are believed to protect phage DNA from host restriction enzymes [9,10,11,12]. Phage genomes may possess various sets of queuosine biosynthesis genes. Besides the *folE*, *queD*, *queC*, and *queE* genes, phage genomes contain *dpdA* and *Gat-queC* (instead of *queC)* genes or encode a preQ_0_ transporter [10,11,12,13]. Notably, queuosine biosynthesis genes have been found in genomes of phages infecting both enterobacteria and other Gammaproteobacteria [11,14].

Here, we report for the first time the genome sequence and characteristics of a new *Enterobacter* phage EC151 belonging to the *Siphoviridae* family and encodes the deazaguanine DNA modification pathway.

## 2. Materials and Methods

### 2.1. Bacterial Strains and Cultivation Media

The Enterobacteria strains used in this study were obtained from the Collection of Extremophilic Micro-organisms and Type Cultures (CEMTC) of the Institute of Chemical Biology and Fundamental Medicine Siberian Branch of the Russian Academy of Science (ICBFM SB RAS), Novosibirsk, Russia. These strains were grown in Luria–Bertani medium and on plates containing Luria–Bertani agar (1.5% *w*/*v*). All cultures were grown at 37 °C.

Phage host strain CEMTC 2064 was obtained from the same collection. The strain was identified as *Enterobacter cloacae* by sequencing a 1308-bp fragment of the 16S rRNA gene and a 501 bp internal portion of the *rpoB* gene, as described previously [15]. Sequences of the 16S rRNA and *rpoB* of the investigated strain were deposited in the GenBank database under accession numbers MW980939 and MZ062217, respectively.

### 2.2. Bacteriophage EC151 Isolation, Propagation and Properties

Bacteriophage EC151 was isolated from a human feces sample, and bacteriophage isolation and propagation were performed as described previously [16]. The study was approved by the Local Ethics Committee of the Center for Personalized Medicine in Novosibirsk, Russia; Protocol #2, 12.02.2019.

The bacterial host range for phage EC151 was examined by spotting serial phage dilutions onto freshly prepared lawns of bacteria, as described previously [17]. Eighty bacterial strains from the Collection of Extremophile Microorganisms and Type Cultures of the ICBFM SB RAS were used for host range examination. The list of strains is given in Table S1.

A phage sample was prepared for electron microscopy as described previously [15]. A drop of phage EC151 suspension was adsorbed for 1 min on a copper grid covered with formvar film; the excess liquid was removed, and the grid was contrasted on a drop of 1% uranyl acetate for 5–7 s. An electron micrograph of phage EC151 particles was obtained using a JEM 1400 transmission electron microscope (JEOL, Tokyo, Japan). Digital images were collected using a side-mounted Veleta digital camera (Olympus SIS, Hamburg, Germany).

### 2.3. Phage DNA Isolation, Genome Sequencing, and Cleavage of EC151 DNA with Type II Restriction Endonucleases

Phage DNA was extracted from the phage preparation, as described previously [18]. Briefly, phage particles were precipitated using a PEG/NaCl solution and dissolved in a STM buffer (10 mM NaCl, 50 mM Tris-HCl, pH 8.0, 10 mM MgCl_2_). RNase and DNase (Thermo Fisher Scientific, Waltham, MA, USA) were added to the phage preparation with a final concentration of 5 μg/mL, and the mixture was incubated for 1 h at 37 °C. The phage suspension was then supplemented with EDTA, proteinase K (Thermo Fisher Scientific, Waltham, MA, USA), and SDS to final concentrations of 20 mM, 100–200 μg/mL, and 0.5%, respectively; the mixture was incubated for 3 h at 55 °C. Afterward, phage DNA was purified by phenol/chloroform extraction and subsequent ethanol precipitation.

A paired-end library of phage EC151 DNA was prepared using the Nextera DNA Sample Preparation Kit (Illumina Inc., Foster City, CA, USA). Sequencing was conducted using the MiSeq Benchtop Sequencer and MiSeq Reagent Kit v.1 (2 × 150 base reads) (Illumina Inc.). The genome was assembled de novo by the CLC Genomics Workbench software v.6.0.1 (Qiagen, Venlo, Netherlands) and resulted in one genomic contig with an average coverage of 150.

Recognition sequences for type II restriction endonucleases were found in the EC151 genome using Vector NTI software [19]. EC151 DNA hydrolysis was performed using endonucleases *Acc*65I, *Apa*I, *Dra*I, *Kpn*I, *Sal*I, and *Xma*I (SibEnzyme, Novosibirsk, Russia), 0.3 μg of DNA was incubated with 2 U of endonuclease in the appropriate buffer at 37 °C overnight, and the hydrolyzed DNA profile was revealed using electrophoresis in 1% agarose gel.

### 2.4. Genome Analysis

Putative open reading frames (ORFs) were annotated using RAST [20] and verified manually by checking all of the predicted proteins against the NCBI GenBank protein database. Screening for t-RNA genes was done using tRNAscan-SE [21]. The CGView server [22] was used for comparative analysis of the EC151 genome and genomes of the *Pseudomonas* phage NP1 (NC_031058), *Escherichia* phage 9 g (NC_024146), and *Escherichia* phage Seurat (NC_027378). MAFFT was used to align genomes [23], and the genetic distance between genomes was calculated using MEGA 7.0 software [24]. A comparative proteomic analysis was performed using a Viral Proteomic tree server [25]. Queuosine pathway components were identified using BLASTx and BLASTp searches against the NCBI GenBank protein database and verified using InterProScan software (https://www.ebi.ac.uk/interpro accessed on 1 June 2021). A phylogenetic analysis of the pathway components with those found in other phages was performed as follows: concatenated protein sequences were prepared using BioEdit 7.2 [26], and the resulting sequences were aligned and analyzed using MEGA 7.0.

## 3. Results and Discussion

### 3.1. Phage Characteristics

**Figure 1:**
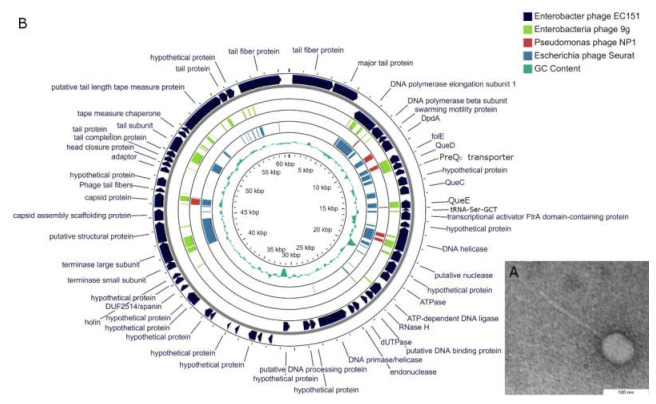
Phage EC151 characteristics. (**A**) Electron micrograph of phage EC151 particle. Transmission electron microscopy, negative staining with 1% uranyl acetate; (**B**) genome map of *Enterobacter* phage EC151 constructed using the CGView server. For sequence similarity comparison, TBLASTX was used versus *Escherichia* phage 9 g (light green), *Pseudomonas* phage NP1 (brown), and *Escherichia* phage Seurat (cyan).

Phage EC151 forms small plaques with a diameter of about 0.5 mm on the lawn of host strain *E. cloacae* CEMTC2064 (Figure S1). Electron microscopy revealed that RP180 has a slightly elongated capsid 62 × 76 nm in diameter, which is connected with a long noncontractile tail of approximately 200 nm in length (Figure 1A). Phage particles’ morphology corresponds to that of *Siphoviridae* family members.

In order to examine the host range of the EC151, a panel of 44 *Enterobacter* strains of seven approved species of the genus and 32 other *Enterobacteriaceae* strains were screened (Table S1). Four *Pantoea agglomerans* strains were included as well. The host range of bacteriophage EC151 was narrow and included only one *E. cloacae* strain (host strain CEMTC 2064) of the 80 tested Enterobacterales strains (Table S1). The host strain had been previously isolated from a clinical sample from a patient with a wound infection.

### 3.2. Genome Characteristics

EC151 genome is a double-stranded DNA molecule with a length of 60,753 bp. The genome contains 58 putative ORFs, 39 of them encoding proteins of predicted function, 18 defined as hypothetical proteins, and one identified as the tRNA-Ser-GCT-encoding gene (Figure 1B). The EC151 nucleotide sequence showed limited similarity to other phage genomes deposited in the NCBI GenBank database, and the BLASTx algorithm revealed the similarity of phage EC151 proteins to proteins of phages belonging to the *Seuratvirus, Nonagvirus*, and *Nipunavirus* genera (Figure 1B) [4,5,6,7]. In addition, the investigated proteins possessed similarities to proteins of many unclassified phages, especially unclassified *Vibrio* phages, and *Pantoea* phage vB_PagS_Vid5 (NC_042120), which is the only representative of the *Vidquintavirus* genus [14]. Note that, among related phages, the tRNA-Ser-GCT-encoding gene was revealed only in the genome of the vB_PagS_Vid5 phage; no tRNA genes were found in the *Nipunavirus, Seuratvirus*, and *Nonagvirus* genomes.

**Figure 2:**
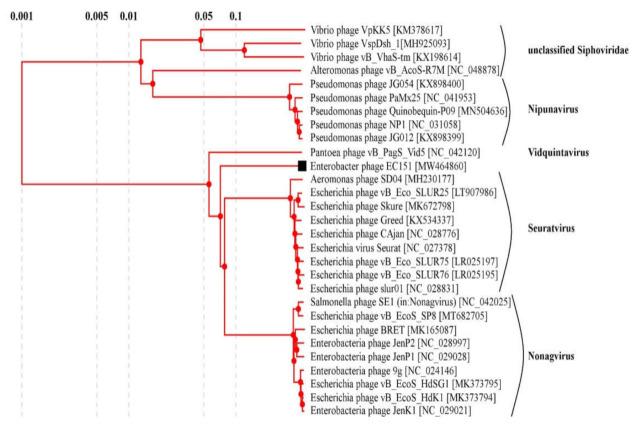
Comparative proteomic analysis of the EC151 with similar bacteriophages prepared using Viral Proteomic tree server. GenBank identifiers (gi) for the sequences are provided in parentheses. The phage EC151 sequence ID is marked by a black box.

The comparative proteomic analysis of the EC151 with similar bacteriophages suggested that the phage EC151 represented a distinct branch within a group of sequences containing clades formed by members of the *Seuratvirus, Nonagvirus*, and *Vidquintavirus* genera (Figure 2). The mean genetic distances in and between these genera and phage EC151 were calculated (Table 1), and it was revealed that the genetic distances between EC151 and the investigated genera were close to the genetic distances between these genera (~0.7 substitutions per site); therefore, EC151 may represent a new genus in the family *Siphoviridae*. The complete genome sequence of *E. cloacae* phage EC151 was deposited in the NCBI GenBank database with the accession number MW464860.

### 3.3. Digestion of EC151 DNA with Type II Restriction Endonucleases

**Figure 3:**
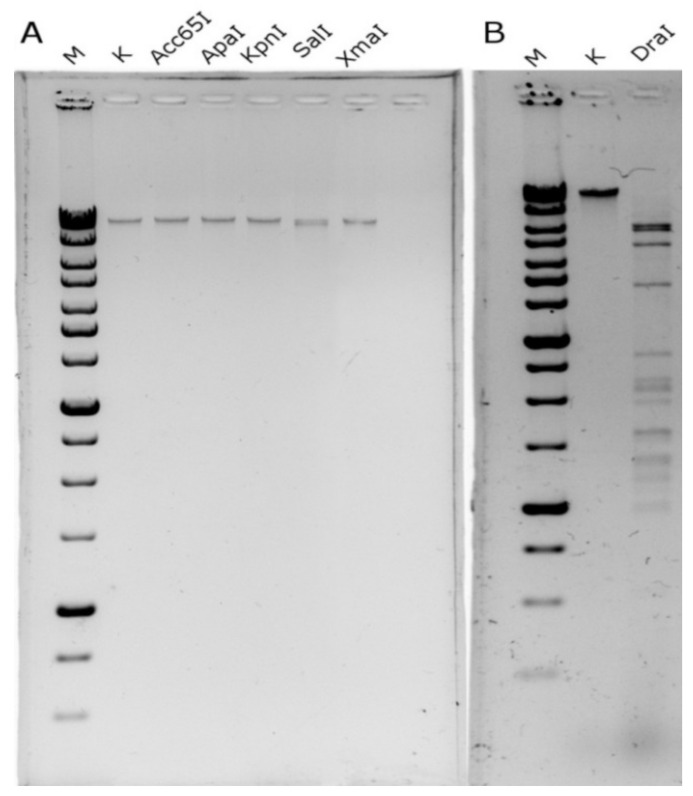
EC151 DNA hydrolysis performed using endonucleases *Acc*65I, *Apa*I, *Kpn*I, *Sal*I, and *Xma*I (**A**) and *Dra*I (**B**). The hydrolyzed DNA profile was revealed using electrophoresis in 1% agarose gel. Lanes: M—DNA molecular weight standard 1kb (SibEnzyme, Novosibirsk, Russia), K—EC151 DNA without endonucleases.

In order to estimate phage EC151 DNA modification, the EC151 genome was screened in silico for recognition sequences of type II restriction endonucleases. *Acc*65I (G^GTACC), *Kpn*I (GGTAC^C), *Apa*I (GGGCC^C), *Dra*I (TTT^AAA), *Sal*I (G^TCGAC), and *Xma*I (C^CCGGG) sites were revealed and chosen for further analysis. The calculated EC151 DNA digestion patterns for these restriction enzymes are summarized in Table S2. It was revealed experimentally that EC151 DNA is highly resistant to hydrolysis with the endonucleases used, except *Dra*I, which is specific to the A/T recognition sequence (TTT^AAA) and hydrolyzes DNA efficiently (Figure 3). Low partial digestion was found in the DNA/*Sal*I hydrolysis, where the recognition sequence contained a G/T cleavage site. All the data suggested that EC151 DNA was highly modified and probably contained G- or C-base modifications.

### 3.4. Queuosine Metabolic Pathway

**Figure 4:**
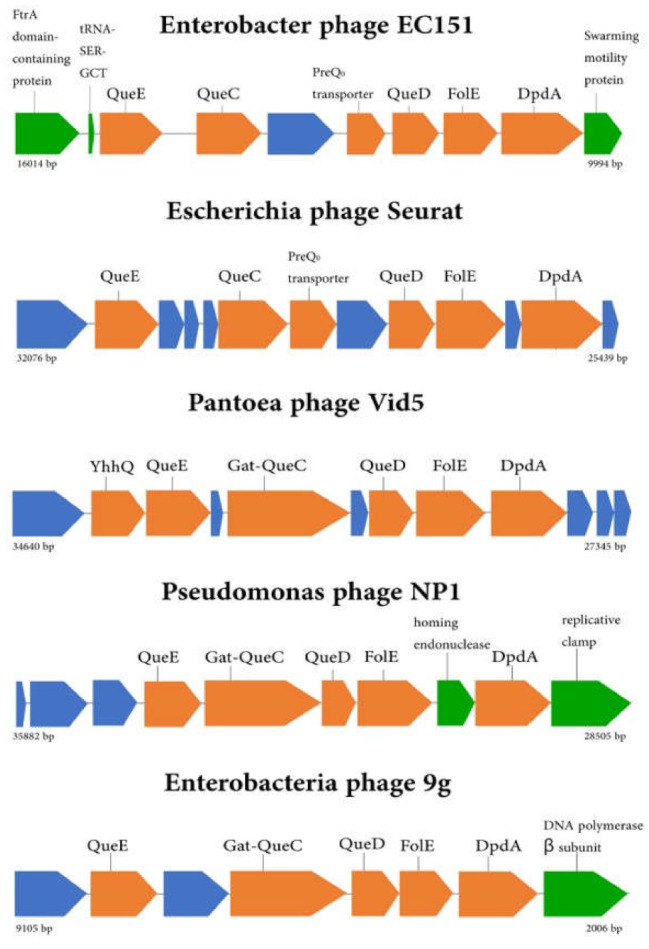
The EC151 deazaguanine DNA modification pathway compared with the corresponding pathways in the most similar phage genomesera. Deazaguanine DNA modification pathway genes are marked with orange boxes, hypothetical proteins are marked with blue, and other proteins are marked with green.

A cluster of genes encoding the deazaguanine DNA modification pathway was revealed in the EC151 genome, similar to that found in the *Nipunavirus, Seuratvirus*, and *Nonagvirus* genera’s genomes [4,5,6,7]. The investigated EC151 genome contains four genes (*folE, queD, queC, queE*), sufficient for the synthesis of a precursor of queuosine (PreQ_0_) and one more gene, *dpdA*, which encodes a DNA-modifying protein (Figure 4). Probably, this phage modifies its DNA with dpreQ_0_, as was shown previously for many phages [11,27]. This cluster contains one more gene, which was annotated using a BLASTx search as a preQ_0_ transporter, and this function was confirmed using InterProScan software. Such proteins are proposed to salvage the preQ_0_ or preQ_1_ precursor from the natural environment [28,29], and phage EC151 probably uses it to increase the preQ_0_ amount in the host bacterial cell.

EC151 is the first *Enterobacter* phage known to contain the full preQ_0_ synthesis pathway; previously, only the *Enterobacter* phage phiEM4 (LC373201) (*Ackermannviridae*; *Agtrevirus*), containing one component of the pathway, the *queC* gene (BBD52218), had been revealed [11].

The EC151 deazaguanine DNA modification pathway was compared with the corresponding pathways of the most similar phage genera (Figure 4). It was found that the EC151 pathway possesses a gene synteny typical of the same cluster of *Seuratvirus* genus members (Figure 4, Escherichia phage Seurat). 

**Figure 5:**
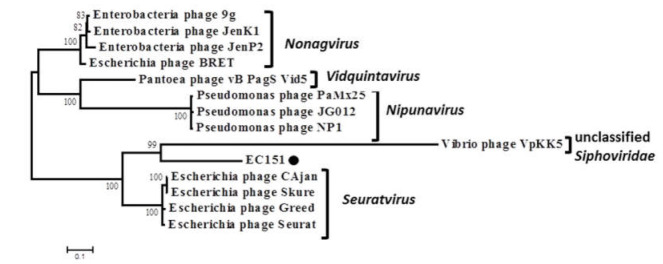
Phylogenetic analysis of the concatenated QueE, QueC, QueD, FolE, and DpdA proteins of the EC151 deazaguanine DNA modification pathway with the most similar protein sequences. Alignment and analysis were performed using MEGA 7.0, and the maximum-likelihood method was applied; bootstrap value 1000 was used. Bayes branch support values above 80% are provided at nodes. GenBank identifiers (gi) for the sequences are provided in parentheses. The *Enterobacter cloacae* phage EC151 sequence is indicated by a black circle.

**Figure 6:**
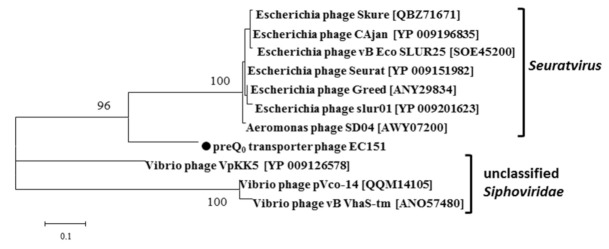
Phylogenetic analysis of the preQ_0_ transporter with the most similar protein sequences. Alignment and analysis were performed using MEGA 7.0, and the maximum-likelihood method was applied; bootstrap value 1000 was used. Bayes branch support values above 80% are provided at nodes. GenBank identifiers (gi) for the sequences are provided in parentheses. The preQ_0_ of *Enterobacter cloacae* phage EC151 is indicated by a black circle.

In order to evaluate the origin of the deazaguanine DNA modification pathway in the EC151 phage, a phylogenetic analysis of the concatenated QueE, QueC, QueD, FolE, and DpdA proteins of this pathway with the most similar protein sequences was performed. It was found that the investigated cluster of proteins grouped with similar proteins of the *Seuratvirus* genus (Figure 5). Note that QueC proteins were found in members of the *Seuratvirus* genus (Figure 4) and some of the unclassified *Vibrio* phages; meanwhile, members of the *Nipunavirus, Vidquintavirus*, and *Nonagvirus* genera encode Gat-QueC proteins (Figure 4), which possess an additional Gat domain required for preQ_0_ modification into the G+ derivative. Proteins similar to the EC151 preQ_0_ transporter were found in *Seuratvirus* genus members and several unclassified phages, especially unclassified *Vibrio* phages. According to the phylogenetic analysis, the EC151 preQ_0_ transporter formed a distinct branch within the clade, which included the members of the *Seuratvirus* genus (Figure 6). Therefore, we can conclude that the deazaguanine DNA modification pathway in the EC151 genome is related to the same pathway in the *Seuratvirus* genus members, which mostly include *Escherichia* phages. Phage EC151 and phages of the *Seuratvirus* genus probably had a common ancestor from which they inherited this operon.

## 4. Conclusions

The novel *Enterobacter* cloacae phage EC151 was characterized for the first time and is suggested to represent a new genus in the *Siphoviridae* family. Electron microscopy showed that the EC151 phage possesses a siphovirus-like capsid morphology and EC151 genome organization is typical for the *Siphoviridae* family. Although increasingly more studies show that the host range of some phages is a function of the environment rather than evolution, EC151 has a very narrow host range. Our results showed that EC151 can only infect *E. cloacae* CEMTC 2064 strain from 80 tested Enterobacterales strains, including 44 strains of seven *Enterobacter* species.

Notably, the EC151 genome encodes a complete set of proteins of the deazaguanine DNA modification pathway. The only type II restriction endonuclease able to efficiently hydrolyze the EC151 genome is *Dra*I with a cleavage site TTT^AAA. This fact confirmed that the deazaguanine DNA modification gene cluster in the EC151 genome is active and provides modifications sufficient to protect phage DNA from at least some restriction enzymes.

The EC151 deazaguanine DNA modification gene cluster has a clear gene synteny with corresponding gene clusters of the *Seuratvirus* genus and a group of unclassified siphoviruses containing mostly Vibrio phages. The trees constructed for the complete protein sequences and proteins encoded by the deazaguanine DNA gene cluster differ in their topology. However, the similarity of phage EC151 proteins to proteins of phages of the *Seuratvirus* genus containing mostly Escherichia phages was higher than other siphoviruses tested in this study. The obtained data supports the idea that the EC151 deazaguanine DNA modification gene cluster may be inherited from the common enterobacterial phage ancestor and remains active because of its necessity to the EC151 life cycle.

## Figures and Tables

**Table 1:** Mean genetic distances in and between members of *Seuratvirus*, *Nonagvirus, Vidquintavirus* genera, and *Enterobacter* phage EC151 in substitutions per site.

Genus	EC151	*Nonagvirus*	*Seuratvirus*	*Vidquintavirus*
EC151	-	0.765	0.731	0.793
*Nonagvirus*	0.765	0.119	0.609	0.717
*Seuratvirus*	0.731	0.609	0.069	0.697
*Vidquintavirus*	0.793	0.717	0.697	-

## Data Availability

Not applicable.

## Supplementary Materials

The following are available online at https://www.mdpi.com/article/10.3390/v13071372/s1, Figure S1. A photograph of the plaques formed by phage EC151 on the lawn of host strain *Enterobacter cloacae* CEMTC 2064. Table S1: The list of microbial strains tested for sensitivity to phage EC151, the strain host CEMTC 2064 is marked with yellow. Table S2: Calculated digestion patterns of EC151 DNA for type II restriction endonucleases. Recognition sequences for endonucleases were found in the EC151 genome using Vector NTI software.

## References

[1] Hormaeche E., Edwards P.R. (1960). A Proposed Genus Enterobacter. Int. J. Syst. Bacteriol..

[2] Davin-Regli A., Lavigne J.-P., Pagès J.-M. (2019). Enterobacter spp.: Update on Taxonomy, Clinical Aspects, and Emerging Antimicrobial Resistance. Clin. Microbiol. Rev..

[3] Mezzatesta M.L., Gona F., Stefani S. (2012). Enterobacter cloacaecomplex: Clinical impact and emerging antibiotic resistance. Futur. Microbiol..

[4] Kulikov E.E., Golomidova A.K., Letarova M.A., Kostryukova E.S., Zelenin A.S., Prokhorov N.S., Letarov A.V. (2014). Genomic Sequencing and Biological Characteristics of a Novel *Escherichia Coli* Bacteriophage 9g, a Putative Representative of a New Siphoviridae Genus. Viruses.

[5] Doan D.P., Lessor L.E., Hernandez A.C., Everett G.F.K. (2015). Complete Genome Sequence of Enterotoxigenic *Escherichia coli* Siphophage Seurat. Genome Announc..

[6] Carstens A.B., Kot W., Lametsch R., Neve H., Hansen L.H. (2016). Characterisation of a novel enterobacteria phage, CAjan, isolated from rat faeces. Arch. Virol..

[7] Flores V., Sepúlveda-Robles O., Cazares A., Kameyama L., Guarneros G. (2017). Comparative genomic analysis of Pseudomonas aeruginosa phage PaMx25 reveals a novel siphovirus group related to phages infecting hosts of different taxonomic classes. Arch. Virol..

[8] Hutinet G., Swarjo M.A., De Crécy-Lagard V. (2017). Deazaguanine derivatives, examples of crosstalk between RNA and DNA modification pathways. RNA Biol..

[9] Weigele P., Raleigh E.A. (2016). Biosynthesis and Function of Modified Bases in Bacteria and Their Viruses. Chem. Rev..

[10] Tsai R., Corrêa I.R., Xu M.Y., Xu S.-Y. (2017). Restriction and modification of deoxyarchaeosine (dG+)-containing phage 9 g DNA. Sci. Rep..

[11] Hutinet G., Kot W., Cui L., Hillebrand R., Balamkundu S., Gnanakalai S., Neelakandan R., Carstens A.B., Lui C.F., Tremblay D. (2019). 7-Deazaguanine modifications protect phage DNA from host restriction systems. Nat. Commun..

[12] Ma R., Lai J., Chen X., Wang L., Yang Y., Wei S., Jiao N., Zhang R. (2021). A Novel Phage Infecting Alteromonas Represents a Distinct Group of Siphophages Infecting Diverse Aquatic Copiotrophs. mSphere.

[13] Crippen C.S., Lee Y.-J., Hutinet G., Shajahan A., Sacher J., Azadi P., de Crécy-Lagard V., Weigele P.R., Szymanski C.M. (2019). Deoxyinosine and 7-Deaza-2-Deoxyguanosine as Carriers of Genetic Information in the DNA of Campylobacter Viruses. J. Virol..

[14] Šimoliūnas E., Šimoliūnienė M., Kaliniene L., Zajančkauskaitė A., Skapas M., Meškys R., Kaupinis A., Valius M., Truncaitė L. (2018). Pantoea Bacteriophage vB_PagS_Vid5: A Low-Temperature Siphovirus That Harbors a Cluster of Genes Involved in the Biosynthesis of Archaeosine. Viruses.

[15] Morozova V., Babkin I., Kozlova Y., Baykov I., Bokovaya O., Tikunov A., Ushakova T., Bardasheva A., Ryabchikova E., Zelentsova E. (2019). Isolation and Characterization of a Novel Klebsiella pneumoniae N4-like Bacteriophage KP8. Viruses.

[16] Morozova V., Fofanov M., Tikunova N., Babkin I., Morozov V.V., Tikunov A. (2020). First crAss-Like Phage Genome Encoding the Diversity-Generating Retroelement (DGR). Viruses.

[17] Kutter E. (2009). Phage Host Range and Efficiency of Plating. Adv. Struct. Saf. Stud..

[18] O’Flaherty S., Coffey A., Edwards R., Meaney W., Fitzgerald G.F., Ross R. (2004). Genome of Staphylococcal Phage K: A New Lineage of Myoviridae Infecting Gram-Positive Bacteria with a Low G+C Content. J. Bacteriol..

[19] Lu G. (2004). Vector NTI, a balanced all-in-one sequence analysis suite. Brief. Bioinform..

[20] Aziz R.K., Bartels D., Best A.A., DeJongh M., Disz T., Edwards R.A., Formsma K., Gerdes S., Glass E.M., Kubal M. (2008). The RAST Server: Rapid annotations using subsystems technology. BMC Genom..

[21] Lowe T.M., Chan P.P. (2016). tRNAscan-SE On-line: Integrating search and context for analysis of transfer RNA genes. Nucleic Acids Res..

[22] Grant J.R., Stothard P. (2008). The CGView Server: A comparative genomics tool for circular genomes. Nucleic Acids Res..

[23] Katoh K., Rozewicki J., Yamada K.D. (2019). MAFFT online service: Multiple sequence alignment, interactive sequence choice and visualization. Brief. Bioinform..

[24] Kumar S., Stecher G., Tamura K. (2016). MEGA7: Molecular Evolutionary Genetics Analysis Version 7.0 for Bigger Datasets. Mol. Biol. Evol..

[25] Nishimura Y., Yoshida T., Kuronishi M., Uehara H., Ogata H., Goto S. (2017). ViPTree: The viral proteomic tree server. Bioinformatics.

[26] Hall T.A. (1999). BioEdit: A user-friendly biological sequence alignment editor and analysis program for Windows 95/98/NT. Nucleic Acids Symp. Ser..

[27] Kot W., Olsen N.S., Nielsen T.K., Hutinet G., De Crécy-Lagard V., Cui L., Dedon P.C., Carstens A.B., Moineau S., Swairjo M.A. (2020). Detection of preQ0 deazaguanine modifications in bacteriophage CAjan DNA using Nanopore sequencing reveals same hypermodification at two distinct DNA motifs. Nucleic Acids Res..

[28] Rodionov D.A., Hebbeln P., Eudes A., ter Beek J., Rodionova I.A., Erkens G.B., Slotboom D.J., Gelfand M., Osterman A.L., Hanson A.D. (2009). A Novel Class of Modular Transporters for Vitamins in Prokaryotes. J. Bacteriol..

[29] Yuan Y., Zallot R., Grove T.L., Payan D.J., Martin-Verstraete I., Šepić S., Balamkundu S., Neelakandan R., Gadi V.K., Liu C.-F. (2019). Discovery of novel bacterial queuine salvage enzymes and pathways in human pathogens. Proc. Natl. Acad. Sci. USA.

