# Supplementary material for: A New Enterobacter cloacae Bacteriophage EC151 Encodes the Deazaguanine DNA Modification Pathway and Represents a New Genus within the Siphoviridae Family
Source: Viruses. 2021 Jul 15;13(7):1372. doi: 10.3390/v13071372 (PMC8310023; doi:10.3390/v13071372)
Supplement: Supplementary file 1 [file viruses-13-01372-s001.zip › Table S1 final.pdf]

№	Species	CEMTC* Strain Number	Isolation source
1	*Enterobacter asburiae*	CEMTC 1100	natural
2	*Enterobacter asburiae*	CEMTC 1123	natural
3	*Enterobacter asburiae*	CEMTC 1229	natural
4	*Enterobacter asburiae*	CEMTC 1622	clinical
5	*Enterobacter asburiae*	CEMTC 1687	clinical
6	*Enterobacter cancerogenus*	CEMTC 3998	insect
7	*Enterobacter cancerogenus*	CEMTC 4000	insect
8	*Enterobacter cancerogenus*	CEMTC 4006	insect
9	*Enterobacter cloacae*	CEMTC 281	clinical
10	*Enterobacter cloacae*	CEMTC 1436	veterinary
11	*Enterobacter cloacae*	CEMTC 1763	clinical
12	*Enterobacter cloacae*	CEMTC 2064	clinical
13	*Enterobacter cloacea*	CEMTC 2083	clinical
14	*Enterobacter cloacae*	CEMTC 2668	clinical
15	*Enterobacter cloacae*	CEMTC 3874	clinical
16	*Enterobacter cloacae*	CEMTC 3929	clinical
17	*Enterobacter cowanii*	CEMTC 1799	clinical
18	*Enterobacter cowanii*	CEMTC 2520	clinical
19	*Enterobacter cowanii*	CEMTC 2691	clinical
20	*Enterobacter cowanii*	CEMTC 2692	clinical
21	*Enterobacter hormaechei*	CEMTC 279	clinical
22	*Enterobacter hormaechei*	CEMTC 1643	clinical
23	*Enterobacter hormaechei*	CEMTC 1719	clinical
24	*Enterobacter hormaechei*	CEMTC 2316	clinical
25	*Enterobacter hormaechei*	CEMTC 2387	natural
26	*Enterobacter hormaechei*	CEMTC 2800	insect
27	*Enterobacter hormaechei*	CEMTC 2801	insect
28	*Enterobacter hormaechei*	CEMTC 2802	insect
29	*Enterobacter hormaechei*	CEMTC 2803	insect
30	*Enterobacter hormaeche*	CEMTC 2804	insect
31	*Enterobacter hormaechei*	CEMTC 2911	veterinary
32	*Enterobacter hormaechei*	CEMTC 3050	clinical
33	*Enterobacter hormaechei*	CEMTC 3751	clinical
34	*Enterobacter hormaechei*	CEMTC 3783	clinical
35	*Enterobacter hormaechei*	CEMTC 3907	clinical
36	*Enterobacter ludwigii*	CEMTC 86	clinical
37	*Enterobacter ludwigii*	CEMTC 87	clinical
38	*Enterobacter ludwigii*	CEMTC 830	clinical
39	*Enterobacter ludwigii*	CEMTC 3805	clinical
40	*Enterobacter xiangfangensis*	CEMTC 2521	clinical
41	*Enterobacter xiangfangensis*	CEMTC 2584	clinical
42	*Enterobacter xiangfangensis*	CEMTC 2928	veterinary
43	*Enterobacter* sp.	CEMTC 2018	plant
44	*Enterobacter* sp.	CEMTC 2035	clinical

45	*Escherichia coli*	ATCC 25922	ATCC
46	*Escherichia coli*	CEMTC 69	clinical
47	*Escherichia coli*	CEMTC 76	clinical
48	*Escherichia coli*	CEMTC 77	clinical
49	*Escherichia coli*	CEMTC 83	clinical
50	*Escherichia coli*	CEMTC 83	clinical
52	*Escherichia coli*	CEMTC 837	clinical
51	*Escherichia coli*	CEMTC 829	clinical
53	*Escherichia coli*	CEMTC 3002	clinical
54	*Escherichia coli*	CEMTC 3017	veterinary
55	*Klebsiella aerogenes*	CEMTC 2420	clinical
56	*Klebsiella aerogenes*	CEMTC 2531	clinical
57	*Klebsiella aerogenes*	CEMTC 2890	clinical
58	*Klebsiella aerogenes*	CEMTC 3530	clinical
59	*Klebsiella grimontii*	CEMTC 1801	clinical
60	*Klebsiella grimontii*	CEMTC 1838	clinical
61	*Klebsiella oxytoca*	ATCC 49131	ATCC
62	*Klebsiella pneumoniae*	ATCC 13883	ATCC
63	*Klebsiella pneumoniae*	ATCC 700603	ATCC
64	*Klebsiella pneumoniae*	CEMTC 270	clinical
65	*Klebsiella pneumoniae*	CEMTC 356	clinical
66	*Klebsiella pneumoniae*	CEMTC 1819	clinical
67	*Klebsiella pneumoniae*	CEMTC 1922	veterinary
68	*Klebsiella pneumoniae*	CEMTC 1967	clinical
69	*Klebsiella pneumoniae*	CEMTC 2067	clinical
70	*Klebsiella pneumoniae*	CEMTC 2071	clinical
71	*Klebsiella pneumoniae*	CEMTC 2274	veterinary
72	*Klebsiella pneumoniae*	CEMTC 2291	clinical
73	*Salmonella enterica*	CEMTC 836	veterinary
74	*Salmonella enterica*	CEMTC 1844	veterinary
75	*Salmonella enterica*	CEMTC 2842	veterinary
76	*Shigella dysenteriae*	CEMTC 2398	clinical
77	*Pantoea agglomerans*	CEMTC 1637	plant
78	*Pantoea agglomerans*	CEMTC 1958	clinical
79	*Pantoea agglomerans*	CEMTC 3028	veterinary
80	*Pantoea agglomerans*	CEMTC 3939	insect

*CEMTC - Collection of Extremophilic Microorganisms and Type Cultures of the Institute of Chemical Biology and Fundamental Medicine,seven approved spesies Siberian Branch of Russian Academy of Science
