# Supplementary material for: A New Enterobacter cloacae Bacteriophage EC151 Encodes the Deazaguanine DNA Modification Pathway and Represents a New Genus within the Siphoviridae Family
Source: Viruses. 2021 Jul 15;13(7):1372. doi: 10.3390/v13071372 (PMC8310023; doi:10.3390/v13071372)
Supplement: Supplementary file 1 [file viruses-13-01372-s001.zip › Table S2 final.pdf]

Table S2. Calculated digestion patterns of EC151 DNA for type II restriction endonucleases

	Acc65I (G^GTACC)	ApaI (GGGCC^C)	DraI (TTT^AAA)	KpnI (GGTAC^C)	SalI (G^TCGAC)	XmaI (C^CCGGG)
Calculated size of DNA fragments (b.p)	18744	29330	9428	18744	18644	31431
	13000	21664	7239	13000	13525	23753
	10526	9760	7190	10526	13133	5570
	5458		4543	5458	7532	
	5283		2975	5283	2425	
	3953		2649	3953	1523	
	3454		2218	3454	1518	
	336		2127	336	1101	
			2090		756	
			1939		389	
			1600		208	
			1596			
			1528			
			1363			
			1360			
			1339			
			1303			
			1238			
			1203			
			1181			
			1100			
			1012			
			549			
			540			
			422			
			320			
			237			
			229			
			190			
			40			
			6			
