# Supplementary figures and images for: A New Enterobacter cloacae Bacteriophage EC151 Encodes the Deazaguanine DNA Modification Pathway and Represents a New Genus within the Siphoviridae Family
Source: Viruses. 2021 Jul 15;13(7):1372. doi: 10.3390/v13071372 (PMC8310023; doi:10.3390/v13071372)

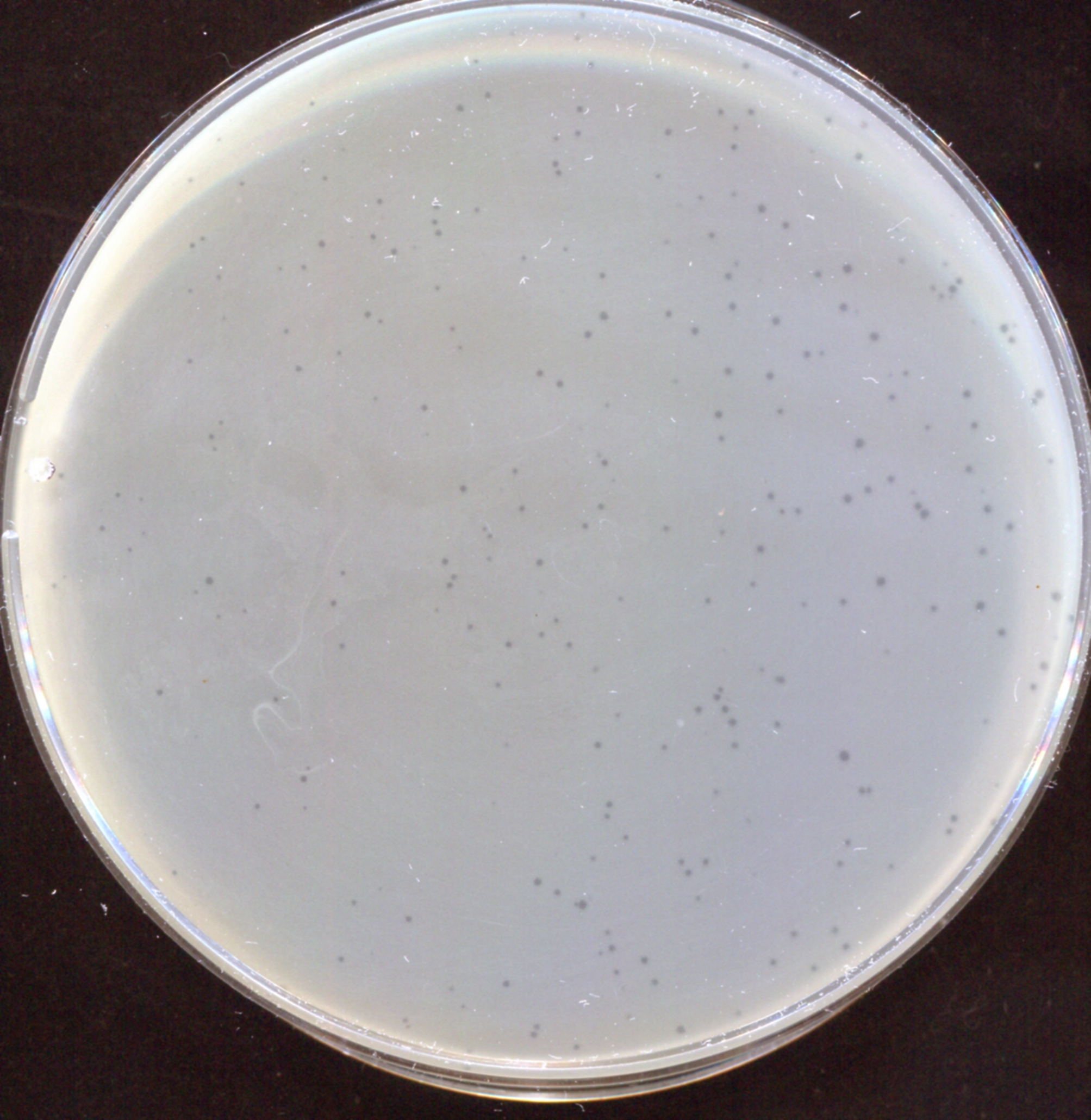

Supplement: Supplementary file 1 [file viruses-13-01372-s001.zip › Figure S1.jpg]
